# Association between HRP–2/pLDH rapid diagnostic test band positivity and malaria–related anemia at a peripheral health facility in Western Uganda

**DOI:** 10.7189/jogh.05.020402

**Published:** 2015-12

**Authors:** Ross Boyce, Raquel Reyes, Moses Ntaro, Edgar Mulogo, Michael Matte, Yap Boum, Mark J. Siedner

**Affiliations:** 1 Department of Medicine, Massachusetts General Hospital, Boston, MA, USA; 2 Department of Community Health, Mbarara University of Science & Technology, Mbarara, Uganda; 3 Epicentre Mbarara Research Center, Mbarara, Uganda; 4 Center for Global Health, Massachusetts General Hospital, Boston, MA, USA

## Abstract

The detection of severe malaria in resource–constrained settings is often difficult because of requirements for laboratory infrastructure and/or clinical expertise. The aim of this study, therefore, was to explore the utility of a multiple antigen (HRP–2/pLDH) rapid diagnostic test (RDT) as a low–cost, surrogate marker of patients at high risk for complications of severe malaria. We reviewed programmatic data at a peripheral health center in Western Uganda. Available demographic and clinical data on all individuals presenting to the center who underwent an RDT for suspected malaria infection were reviewed. We fit logistic regression models to identify correlates of two outcomes of interest: 1) severe malaria–related anemia, defined here as hemoglobin ≤7g/dL and 2) receipt of parenteral quinine. 1509 patients underwent malaria testing with an SD FK60 RDT during the observation period. A total of 637 (42%) RDTs were positive for at least one species of malaria, of which 326 (51%) exhibited a single HRP–2 band and 307 (48%) exhibited both HRP–2 and pLDH bands, while 4 exhibited only a single pLDH band. There was a trend towards more severe anemia in patients with a HRP–2/pLDH positive RDT compared to a HRP–2 only RDT (β = –0.99 g/dl, 95% CI –1.99 to 0.02, *P* = 0.055). A HRP–2/pLDH positive RDT was associated with an increased risk of severe malaria–related anemia compared to a negative RDT (adjusted odds ratio (AOR) 18.8, 95% CI 4.32 to 82.0, *P* < 0.001) and to a HRP–2 only RDT (AOR 2.46, 95% CI 0.75 to 8.04, *P* = 0.14). There was no significant association between RDT result and the administration of parenteral quinine. These results offer preliminary evidence that specific patterns of antigen positivity on RDTs could be utilized to identify patients at an increased risk for complications of severe malaria.

Each year millions of African children are infected with *P. falciparum* malaria, and approximately 550 000 die from manifestations of severe disease [1]. In areas of high transmission intensity, severe anemia is the most common complication of severe malaria, which primarily affects infants and young children [2]. While delays in the diagnosis and treatment of malaria are associated with poor outcomes [3-5], effective management requires well–equipped hospitals with highly trained clinicians, which are lacking in many malaria endemic settings [6,7]. Mortality among children with severe malaria anemia who receive care at hospitals and referral centers has been reported as high as 10–20% [8-11], and it may be even higher among those presenting to peripheral health facilities, where human resources and acute care services, including access to mechanical ventilation, hemodialysis, and blood transfusion, are limited [12,13].

A major challenge to the identification of severe malaria stems from the rigorous laboratory–based diagnostic criteria [2,14,15]. Hemoglobin, glucose, creatinine, and lactate levels are rarely available at peripheral health facilities in resource–constrained settings [14,15]. Even light microscopy is often inaccessible because of the requisite infrastructure and technical expertise [7,13,16,17]. Without proper diagnostic tools, health care workers may misidentify and inappropriately triage cases of severe malaria [7]. An ideal diagnostic test would enable health care workers, especially nurses, midwives, and community health workers, to accurately and quickly identify patients either with or at risk for complications of severe malaria and initiate expedited management practices.

Rapid diagnostic tests (RDTs) are low–cost, simple tools for the diagnosis of malaria and are particularly valuable in settings where microscopy is not readily available [18-21]. Newer generation RDTs that incorporate multiple antigens on a single membrane have been developed to differentiate between various *Plasmodium* species infections. Additionally, these RDTs have shown some ability to provide a quantitative estimate of parasite density due to the differing sensitivities of the respective antigens [22-24]. Because parasite density can be a correlate of disease severity, the use of multiple antigens on a single membrane has the potential to aid in detection of severe malaria. No study, however, has examined the association of RDT band positivity with clinical parameters. Thus, we reviewed data from a peripheral health center in rural Uganda to assess for a correlation between band RDT positivity and markers of disease severity. Our overarching goal was to explore the potential use of a multiple–antigen RDT as a low–cost, scalable marker of patients at risk of severe malaria–related anemia.

## METHODS

### Study site

We evaluated programmatic data during the period January–March 2013, collected from the Bugoye Level III Health Center (BHC), in the Kasese District of Western Uganda (0° 18’ North, 30° 5′ East). The health center functions as a referral center for the Bugoye sub–county, and serves a rural population of approximately 50 000 residents. The center houses a small laboratory with staff trained to perform light microscopy and RDTs, as well other basic tests, such as hemoglobin estimation.

### Study overview

The objective of this retrospective, observational pilot study was to assess the diagnostic utility of a three–band (HRP–2/pLDH) RDT at a resource–limited, rural health center in malaria–endemic Western Uganda.

### Study procedures

We reviewed data on all individuals with fever who underwent an RDT for suspected malaria infection. We abstracted data on patient age, sex, village of residence, patient disposition, laboratory results, and medication prescriptions from clinic registers.

### Laboratory procedures

Laboratory diagnosis of malaria was made using the Standard Diagnostics FK60 Malaria Ag *P. falciparum*/Pan RDT (Standard Diagnostics, Hagal–Dong, Korea). The RDT is a validated antigen detection test with three individual bands signifying the control, the HRP–2, and the pLDH antigens [23,25,26]. The presence of a control band in the absence of either a HRP–2 or pLDH result indicates a negative test. The presence of a single HRP–2 line denotes infection with *P. falciparum,* whereas a unique pLDH line indicates infection with one or more of the other *Plasmodium* species. The presence of a positive HRP–2 line together with a pLDH line indicates an infection with either *P. falciparum* or a mixed–species infection.

RDTs (Lot #090192) for the study were obtained from Kampala Pharmaceutical Industries (Kampala, Uganda), and stored in their original packaging at room temperature. RDTs were performed and interpreted by two members of the BHC laboratory staff in accordance with the manufacturer’s instructions. In cases in which the control line did not appear, the result was considered invalid and the test was repeated. Faint HRP–2 and pLDH test lines were considered positive.

When available, we reviewed matched thick and thin smears for specimens with three–band positive tests. Smears were prepared using a modified Field’s Stain and examined by light microscopy [27]. Asexual parasitemia of any level was reported as a positive smear. Blood smears were transported to the Epicentre Mbarara Research Center for confirmatory review, where two expert microscopists, who were blinded to the field results, independently read the slides. Hemoglobin (Hb) levels were estimated using Sahli’s method [28] as is the standard of care at BHC.

### Statistical analysis

Data were entered into Microsoft Excel (Redmond, WA) and analyzed with Stata 12.1 (College Station, TX, USA). We summarized patient characteristics and compared those with two– and three–band positive RDTs using Pearson chi–squared testing. We fit univariable logistic regression models to identify correlates for two outcomes of interest: 1) severe anemia, defined as a hemoglobin <7 g/deciliter (g/dL) and 2) receipt of parenteral quinine. While the WHO traditionally define severe malaria-related anemia a normocytic anemia of Hb <5 g/dL in children and <7 g/dL in adults, we used hemoglobin values of <7 g/dL as the clinically relevant outcome as it represents the transfusion threshold for severe malaria in adults and approaches the threshold at which transfusion should be considered in children when present with other clinical features [2,29].

Our primary explanatory variable of interest was RDT test result, defined as HRP–2 vs HRP–2/pLDH positive. Secondary explanatory variables included sex, age, village distance from the health center, village elevation, and transmission season, defined as high (January) or low (February, March). We fit multivariable models including all variables that were significant in univariable models with a pre–specified *P*–value of <0.25 [30]. A resulting *P–*value of <0.05 was considered statistically significant in the final models.

### Ethics statement

Ethical approval for study procedures and data collection was provided by the institutional review boards of Partners Healthcare and the Mbarara University of Science and Technology. Informed consent was not required by the ethical review committees due to the programmatic nature of the project.

## RESULTS

During the observation period, 1509 patients underwent malaria testing with an RDT. A total of 637 RDTs (42%) were positive for malaria. Of the positive RDTs, 326 (51.2%) exhibited a single HRP–2 band, 307 RDTs (48.2%) exhibited both HRP–2 and pLDH bands, while only 4 RDTs (0.6%) exhibited a single pLDH band. Characteristics of the study sample, stratified by RDT result, are summarized in **Table 1**.

**Table 1:** Univariable comparison of patients with positive RDT by antigen category

Characteristic	HRP–2 positive	HRP–2/pLDH positive	*P*–value
**Number (n, %)**	326 (51.5)	307 (48.5)	–
**Sex (Male/Female)**	124/202	123/184	0.60
**Age (median, interquartile range):**	31 (15, 58)	25 (12, 58)	0.22
<5 years (n, %)	87 (26.7)	87 (28.3)	0.64
<15 years (n, %)	192 (58.9)	216 (70.4)	0.003
**Village (n, %):**			
distant from Clinic	22 (6.8)	18 (5.9)	0.69
dow elevation	152 (46.6)	141 45.9)	0.03
**Transmission season:**			
high (n, %)	119 (36.5)	164 (53.4)	<0.001
low (n, %)	207 (63.5)	143 (46.6)	

Notably, the prevalence rate of HRP–2/pLDH positive results was twice as high among patients <15 years compared to those ≥15 years. The absolute number and proportion of HRP–2/pLDH positive RDTs declined during the transition from high to low transmission seasons, while that of HRP–2 only positive RDTs remained relatively stable.

Of the 307 patients with HRP–2/pLDH positive RDTs, 90 (29.3%) had corresponding blood smears available for interpretation. The majority (83/90, 92.2%) of smears from patients with HRP–2/pLDH positive RDT results demonstrated *P. falciparum* mono–infections, although there were 4 mixed–species infections (2 *Pf/Pv*, 1 *Pf/Po*, 1 *Pf/Pm*). In addition, there were two non–*P. falciparum* mono–infections (1 *Po*, 1 *Pm*), which may have represented sub–patent *P. falciparum* co–infection not seen on microscopy. All four of the pLDH–only positive RDTs were identified as *P. ovale* mono*–*infections. Parasite density was reported for 89 of 90 of the three–band positive individuals. The mean density was 73 105 parasites/μL with a median density of 40 200 parasites/μL (IQR = 12 100–103 238). Parasite densities were only available for three patients with HRP–2 positive RDTs.

The sensitivity for *P. falciparum* infections (including mixed–species infections) was 99.1% (95% CI 94.5% to 99.9%). The specificity was 75% (95% CI 53.0% to 89.4%), although only a small number of smears were prepared in patients with paired HRP–2 positive (n = 19) and negative (n = 19) RDTs. If, however, we assume that the two RDTs that were three–band positive but negative on microscopy represented true *P. falciparum* infections, as seen when confirmed by PCR in other studies [31,32], the specificity improves to 81.8%.

**Figure 1:**
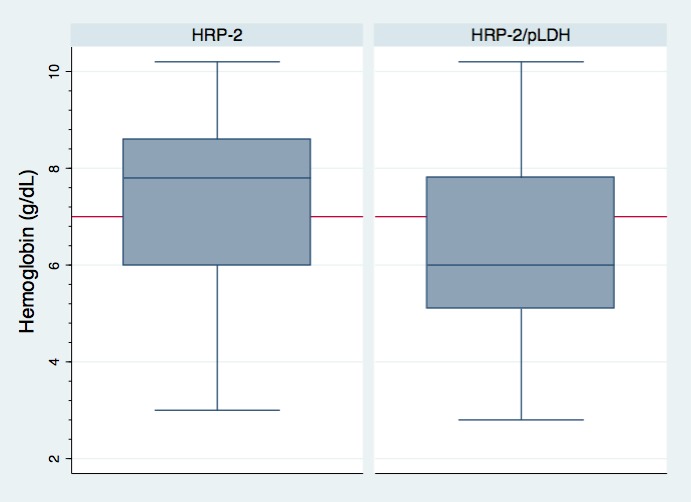
Box plot showing distribution of hemoglobin level in HRP–2 vs HRP–2/pLDH positive rapid diagnostic tests.

During the observation period, a total of 85 paired hemoglobin (Hb) levels were assessed in patients also undergoing an SD FK60 RDT. Children <5 years of age were more likely to have a Hb performed and had a higher proportion of values <7 g/dL (56.4% vs 26.1%) compared to those individuals ≥5 years of age. The mean Hb was 7.3g/dL (95% CI 6.6 to 8.1) in patients with a HRP–2 only positive RDT, and 6.3g/dL (95% CI 5.6 to 7.1) in patients with a three–band positive RDT (β = –0.99 g/dL, 95% CI –1.99 to 0.02, *P* = 0.055, **Figure 1**).

In multivariate logistic regression models, a HRP–2/pLDH positive RDT was the strongest predictor of severe anemia (**Table 2**). The odds of severe malaria anemia were more than double in those patients with a HRP–2/pLDH positive RDT compared to those with an HRP–2 only positive RDT (AOR 2.46, 95% CI 0.75 to 8.04, *P* = 0.14) with a trend toward statistical significance.

**Table 2:** Logistic regression models for correlates of hemoglobin <7 mg/dL

	Univariable model			Multivariate model		
**Characteristic**	**OR**	**95% CI**	***P*–value**	**AOR**	**95% CI**	***P*–value**
**Male sex**	1.51	0.62–3.72	0.37	–	–	–
**Age <15 years**	4.47	1.49–13.38	0.007	4.55	1.32–15.6	0.016
**High season**	0.85	0.35–2.05	0.72	–	–	–
**RDT result:**						
negative	Reference			Reference		
HRP–2 only	7.69	1.81–32.6	0.006	7.49	1.70–33.1	0.008
HRP–2/pLDH	18.0	4.37–74.2	<0.001	18.8	4.32–82.0	<0.001

OR – odds ratio, CI – confidence interval, AOR – adjusted odds ratio, RDT – rapid diagnostic test, HRP–2 – histidine rich protein–2, pLDH – pan–lactate dehydrogenase.

*Explanatory variables with a *P*–value of <0.25 in the univariable model were included in the multivariable model.

We identified 179 patients who were admitted to the inpatient ward with an admission diagnosis of malaria. RDT results were available for 116 (64.8%) of these patients. There were no differences in rates of inpatient admission between those with HRP–2 only and HRP–2/pLDH positive RDTs. Patients with HRP–2/pLDH positive results were more likely to receive treatment with parenteral quinine and anti–pyretics. In the multivariate analysis for correlates of receipt of parenteral quinine, the strongest associations were seen with three–band positive RDT and infection during high transmission season, although neither of these reached statistical significance (**Table 3**).

**Table 3:** Logistic regression models for correlates of parenteral quinine

	Univariable model			Multivariate model		
**Characteristic**	**OR**	**95% CI**	***P*–value**	**AOR**	**95% CI**	***P*–value**
**Male sex**	1.47	0.43–4.98	0.54	–	–	–
**Age <15 years**	0.89	0.25–3.19	0.85	–	–	–
**Comorbidity**	3.44	0.81–2.43	0.35	–	–	–
**Antibiotics**	0.63	0.18–2.26	0.48	–	–	–
**High season**	6.52	0.80–52.9	0.079	5.59	0.68–46.0	0.11
**3–band RDT**	2.36	0.66–8.43	0.19	1.92	0.52–7.06	0.33

OR – odds ratio, CI – confidence interval, AOR – adjusted odds ratio, RDT – rapid diagnostic test, HRP–2 – histidine rich protein–2, pLDH – pan–lactate dehydrogenase.

*Explanatory variables with a *P*–value of <0.25 in the univariable model were included in the multivariable model.

## DISCUSSION

Our results show a trend towards patients with HRP–2/pLDH positive RDTs having more severe malaria–related anemia that those patients with an HRP–2 only positive RDT. While previous work with the SD FK60 in a reference laboratory found a correlation between parasite density and the presence of the pLDH band [23], our findings linking three–band positivity to anemia are novel. Our findings provide preliminary evidence that multiple band RDTs, with antigens of differing sensitivity, may have the potential to serve as an adjunctive tool for the identification of patients at high risk of severe malaria–related anemia. Further studies are needed to determine if incorporating three–band positivity into malaria case management algorithms at peripheral health facilities might accurately identify patients either with or at risk for complications of severe malaria.

We postulate that the positive pLDH band, which is less sensitive than HRP–2, is a marker of higher parasite density [33-35]. Findings from our study that would support this association include: 1) nearly a quarter of three–band positive smears demonstrated parasite densities >100 000/μl; 2) a higher prevalence of three–band positive RDTs among individuals <15 years of age who have not yet acquired protective immunity [36-39]; 3) high relative rates of three–band positive disease during the traditional high transmission season; and 4) the higher degree of anemia among patients with a HRP–2/pLDH positive RDT. We note that previous studies have shown that the degree of anemia correlated with both parasitemia and schizontemia [40].

An alternate explanation for the relationship could be HRP–2 antibody persistence leading to false–positive HRP–2 positive RDTs. Prior studies in Uganda have reported HRP–2 band specificities as low as 62% [31]. These false–positive HRP–2 tests could result after recent treatment with antimalarials, making those patients with acute illness as indicated by HRP–2/pLDH band positivity appear relatively ill.

However, our clinical experience in Bugoye suggests that very few patients seek treatment for malaria outside of the government health center, given difficult terrain and high costs associated with private facilities. In settings where there is a low probability of prior treatment, such as our site, the specificity of the HRP–2 antigen is typically higher than that associated with studies conducted at urban referral centers [31,41,42]. As evidence of this, studies from more rural sites in Uganda that have utilized polymerase chain reaction (PCR) to investigate discordant RDT and microscopy results have found the PCR–corrected specificity to be significantly higher [32,43]. Additionally, validation studies with the SD FK 60 in field conditions from other regions have reported a specificity for *P. falciparum* malaria exceeding 95% [26].

Our study, which was hypothesis generating in nature and primarily involved a retrospective review of routinely collected data, has a number of limitations. Foremost among these is the lack of available paired slides for all specimens. The available slides and Hb results were prepared in a non–random manner at the direction of the supervising clinical officer. We did, however, review smears for nearly 30% of those patients with three–band positive RDTs and Hb values for nearly 15% of patients with positive RDTs. The second major limitation of our study was that the use of the Sahlis method for Hb estimation, which while simple and inexpensive, can be prone to dilution and reading errors. We believe there should be minimal bias stemming from this method because errors would be stochastic and a single staff member interpreted all results. Finally, we performed this study at a single site and with a relatively small sample size, which reinforces the need for corroboration of these findings in larger, and more diverse patient populations. A follow up, prospective study is now under way.

## CONCLUSIONS

In summary, our findings suggest that patients with HRP–2/pLDH positive RDTs have more severe malaria–related anemia than patients with HRP–2 only positive RDTs. We postulate that the positive pLDH band, which is less sensitive than HRP–2, is a marker of higher parasite density and correspondingly, more severe anemia. These results offer preliminary evidence that multiple antigen RDTs could be utilized to identify patients at an increased risk for complications of severe malaria. If confirmed, multiple antigen RDTs may play a role in case management algorithms at peripheral health facilities. Further studies with more comprehensive data collection methods, larger sample sizes, and longer timeframes should be pursued before this approach is adopted.
